# Application of 3D Printing Technology in Dentistry: A Review

**DOI:** 10.3390/polym17070886

**Published:** 2025-03-26

**Authors:** Yangqing Chen, Junchao Wei

**Affiliations:** 1 School of Stomatology, Jiangxi Medical College, Nanchang University, Nanchang 330006, China; 13684818053@163.com; 2 School of Chemistry and Chemical Engineering, Nanchang University, Nanchang 330031, China; 3 Jiangxi Provincial Key Laboratory of Oral Disease, Nanchang 330006, China; 4 Jiangxi Province Clinical Research Center for Oral Disease, Nanchang 330006, China

**Keywords:** healthcare, 3D printing, dental medicine, tissue engineering, regeneration

## Abstract

Three-dimensional (3D) printing is a cutting-edge technology that is widely used in biomedical fields to construct various commercial products or scaffolds for theoretical research. In this review, 3D printing technologies with different principles are briefly introduced, including selective laser melting (SLM), selective laser sintering (SLS), fused deposition modeling (FDM), stereolithography (SLA), and digital light processing (DLP). In addition, the applications of 3D printing in dentistry, such as dental implantology, prosthodontics, orthodontics, maxillofacial surgery, and dental tissue regeneration, were summarized. Furthermore, the perspective and challenges of 3D printing were also addressed to help the readers obtain a clear map for the development of 3D printing in dentistry.

## 1. Overview of 3D Printing Technology

3D printing, or additive manufacturing, is an advanced technology that uploads a digital file through a computer to create a 3D solid object [1]. Its basic principle is a layer-by-layer printing rapid manufacturing method. The printing process consists of superimposing the raw materials to construct a 3D object through layer-by-layer printing, forming directly, quickly, and without waste of raw materials [2]. The first 3D printer was invented in 1986. As a promising technology with the rise in advanced fabrication techniques, 3D printing has rapidly evolved over its nearly 40 years of history [3].

3D printing technology has been applied in various fields, such as medicine, automotive, aerospace, electronics, art, and construction [4], and it shows especially great potential in biomedical applications [5]. Three-dimensional printing technology has broad application prospects in the field of medical devices, and it is very rich in material selection. It is suitable for printing various materials, such as metal materials (Ti, Mg, Zn, and some alloys, etc.) [6], inorganic non-metallic materials (calcium apatite, ceramics, etc.) [7], and polymer materials (polylactic acid, polycaprolactone, polyetheretherketone, etc.) [8,9]. The unique feature of 3D printing is a customized design, which facilitates the control of shapes, structures, and special functions. Due to these advantages and features, 3D printing has significant prospects, especially in dentistry, where more precision is required [10].

Nowadays, 3D printing technologies with different working principles, such as selective laser melting (SLM), selective laser sintering (SLS), fused deposition modeling (FDM), stereolithography (SLA), digital light processing (DLP), and so on [11], have been widely investigated and applied in dentistry. Herein, the principles of 3D printing technology are briefly introduced, meantime, the raw materials, advantages, challenges and costs of different 3D printing are listed in Table 1. Besides, the applications of these technologies in dentistry are also concisely summarized.

## 2. Three-Dimensional Printing Technologies of Different Principles

### 2.1. Selective Laser Melting (SLM)

Selective laser melting (SLM) is an additive manufacturing technology that utilizes a high-energy laser beam to selectively melt and solidify metal powder layer by layer, based on 3D CAD models, to produce complex metal parts. This method overcomes traditional machining limitations by enabling the manufacturing of complex internal structures and geometries [12]. SLM has gained significant attraction in various fields due to its high precision, ability to create complex structures, and superior material properties [13]. In dentistry, SLM is employed to fabricate personalized titanium meshes for bone augmentation in implantology, enhancing surgical accuracy and success rates [14]. Additionally, it is widely used to produce high-precision, durable, and esthetically pleasing metal restorations tailored to individual patients’ needs [15]. However, SLM’s reliance on metal raw materials limits its scope. Consequently, the integration of other 3D printing technologies with broader material compatibility is essential to expand its applications in dentistry.

### 2.2. Selective Laser Sintering (SLS)

Selective laser sintering (SLS) is an advanced 3D printing technology that employs a controlled laser beam to sinter or melt powdered materials layer by layer, enabling the fabrication of complex 3D parts [16]. SLS supports a broader range of raw materials, including polymers, metals, ceramics, and their composites, making it suitable for diverse and personalized applications. This versatility allows SLS to produce lightweight, high-precision dental replacement products, such as molds and dentures. However, despite its advantages, the high costs of equipment and materials associated with SLS limit its application to some extent.

### 2.3. Fused Deposition Modeling (FDM)

Fused deposition modeling (FDM) is a 3D printing technology that heats and extrudes thermoplastic materials (such as PLA and nylon) through a thin nozzle, depositing them layer by layer to create solid objects. FDM is cost-effective compared to other 3D printing methods as it utilizes low-cost equipment and materials [17]. In dentistry, FDM is employed to produce personalized dentures, braces, and implants for individual patients, ensuring a high degree of oral structure compatibility. However, further optimization of FDM is required to overcome the limitations, including lower dimensional accuracy, rough surface finishes, and concerns regarding durability and oral safety. Additionally, its inefficiency makes it unsuitable for large-scale production. Nevertheless, FDM remains an economical choice for small-batch production in dental applications.

### 2.4. Stereolithography (SLA)

Stereolithography (SLA) technology is based on a UV laser or other light source to cure a liquid photosensitive resin [18]. During the printing process, the laser beam scans the surface of the liquid resin according to a designed path, solidifies it, and forms a layer, which is repeated layer by layer until the entire model is printed [19]. Since each layer is cured by laser scanning, the printed model surface is usually very smooth and has high accuracy. Therefore, SLA technology can also be used to make medical models, surgical guides, etc., to provide accurate and personalized solutions for patients. With the continuous advancement of technology and the reduction in costs, SLA technology is expected to have bright prospects in dentistry.

### 2.5. Digital Light Processing (DLP)

Digital light processing (DLP) is a light-curing 3D printing technology that utilizes a UV projector to solidify photosensitive polymer liquid layer by layer, enabling the fabrication of high-precision, smooth-surfaced 3D objects [20]. Due to its fast molding speed and exceptional accuracy, DLP is widely used to process materials such as zirconia, alumina, and hydroxyapatite, making it particularly suitable for dental applications [21,22]. In dentistry, DLP is employed to produce surgical guides, invisible braces, dental models, and direct-printed implants. Compared to traditional methods, DLP offer superior precision, biocompatibility, and personalization, simple workflows, and thus have greatly revolutionized the dental industry.

## 3. Application of 3D Printing Technology in Dentistry

Dental health is critical to human beings; however, many dental diseases bring great suffering to humans [23]. Although various clinical technologies and operations have solved these problems to some extent, some serious tissue defects in dentistry still need more precise operation, which involves the application of advanced medical devices or tissue regeneration, needing more advances in modern technology to replace traditional treatments to achieve.

**Figure 1:**
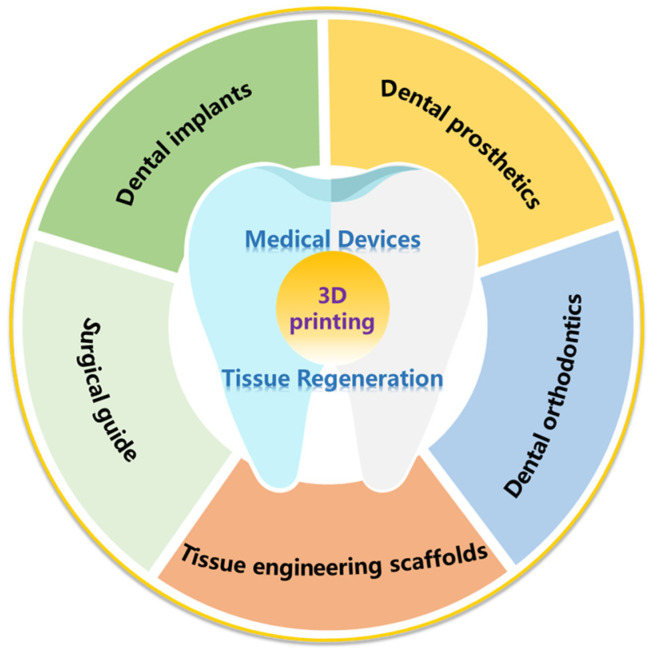
Current applications of 3D printing technology in dentistry.

Hence, 3D printing technology, as a sophisticated and custom-made technology, has great potential in dental applications, for example, dental implants, dental prosthesis, dental orthodontics, maxillofacial surgery, and dental tissue regeneration (Figure 1). Due to the rapid development of 3D printing technology and the diversity of printing raw materials, 3D printing technology is more and more widely used in stomatology.

### 3.1. The Application of 3D Printing Technology in Medical Devices

#### 3.1.1. Surgical Guide

The surgical guide is a personalized surgical aid tool that accurately transfers the preoperative virtual design operation scheme to the patient’s mouth, which is a powerful tool for achieving accurate surgery in clinical practice. Surgical guides are often used to aid the operation of dental implants and maxillofacial surgery, allowing the dentist to place the implant in the safest, most predictable and efficient way, which may help shorten the surgical time, improve the accuracy of surgery, reduce non-traumatic surface damage, and alleviate the pain of patients [24].

**Figure 2:**
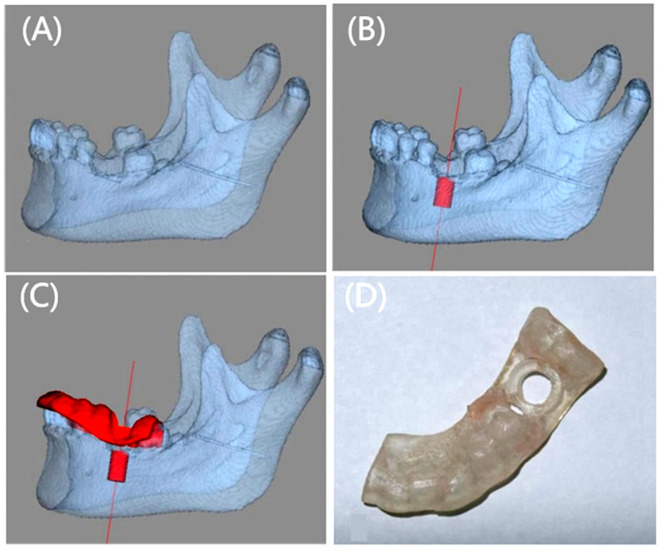
The basic workflow for making a 3D printing surgical guide. (**A**) Digital model of the mandible obtained by scanning. (**B**) Planned implant position in the design software. (**C**) Design surgical guide with the software. (**D**) 3D printed surgical guide. Reprinted with permission from John Wiley and Sons [26].

**Figure 3:**
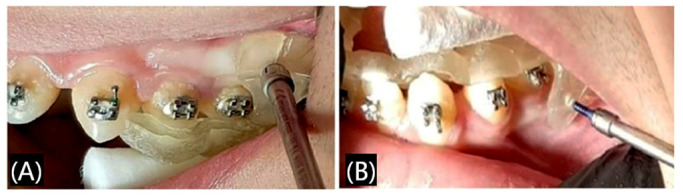
A case of bimaxillary alveolar protrusion treatment with a 3D printed surgical guides. (**A**) Upper jaw. (**B**) Lower jaw. Reprinted with permission from MDPI [28].

Traditional dental implantation requires complex processes, which cause serious damage to native dental tissue, while the emergence of 3D printing guidance technology has greatly simplified the steps in the surgical process [25]. The basic procedure to prepare a 3D printed surgical guide for dental implantation is shown in Figure 2 [26]. Firstly, CBCT scanning is performed to collect the 3D data of the alveolar bone, which may clearly show the 3D environment of the native bone tissue. Secondly, a digital guide template is generated in the software and then used for 3D printing to build a clinical implant guide template. Finally, the doctor placed the implants in the ideal position with the help of a guide template, which can effectively avoid tooth tissue damage and tooth nerve malposition and thus may be helpful for the implant’s long-term stability. Compared with traditional implantation methods, 3D printed surgical guides greatly reduce the number of surgical tools used and improve the accuracy and stability of surgical use [27]. At present, 3D printed surgical guides have been accepted greatly in the clinic and commercial products have been used to help doctors to handle complex operations. Figure 3 shows a case of a 16-year-old male patient with 3D printed guides for bimaxillary alveolar protrusion, which was treated successfully by accurate placement, uprighting the incisors, retracting the anterior dentition, closing upper and lower arch spaces, and improving facial esthetics [28].

#### 3.1.2. Dental Implants

**Figure 4:**
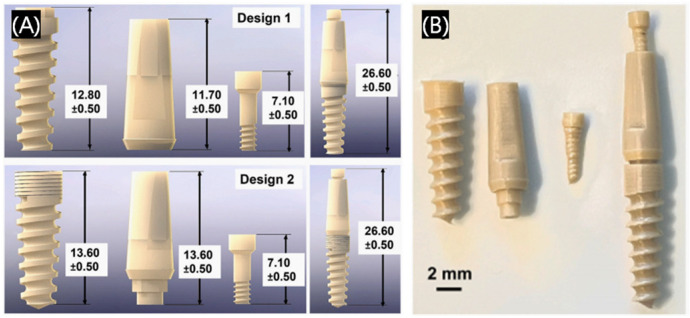
Three-dimensional printed poly-ether-ether-ketone (PEEK) dental implant roots. (**A**) Computer-aided diagram (CAD) drawing showing the design of two dental implant designs for 3D printing. (**B**) Digital photographs of the 3D printed PEEK implant system. Reprinted with permission from Elsevier [29].

Dental implantation is the process of placing the implant into the alveolar bone, dental implants are used to replace missing teeth. A significant demand for dental implants in clinical practice is needed; however, the current problem of low matching degree between the implant and tooth extraction socket is unresolved, which leads the bone combination is difficult to form. Three-dimensional printed personalized root-shaped implants can promote stable bone bonding between the implant and the alveolar bone, and better simulate natural teeth. Figure 4 shows a patient-specific 3D printed poly-ether-ether-ketone (PEEK) dental implant system [29]. Some brands have already used 3D printing technology to produce dental implants and achieved commercialization, so 3D printing dental implants have been relatively widely used and developed in the clinic. The traditional patient-customized implants are gradually replaced by 3D printing implantable medical devices [30].

Since the surface coating of implants will be gradually worn during the use process, due to the complex physiological environment of the body, which cannot maintain long-term efficacy, how to enhance the adhesion between the biological materials and the substrate, and obtain the integrated multifunctional active, good biocompatibility and durability of printed implants is the key issue in the current study [31].

#### 3.1.3. Dental Prosthetics

Tooth loss/fracture is very common phenomenon, which may be caused by aging, accidents, caries, periodontal disease, and some unhealthy lifestyles [32]. Therefore, dental function restoration is necessary, and thus various commercialized tooth substitutes are needed to replace missing natural teeth. However, the traditional method of preparing artificial teeth or prosthetic devices needs complex steps and is a time-consuming process. In addition, sometimes it is difficult to control the exact size or structure to satisfy the patients. Three-dimensional printing technology makes up for the above shortcomings which are confused in dental prosthetics in dentistry, owing to its advantages of a short processing time and the precise design of individual products.

**Figure 5:**
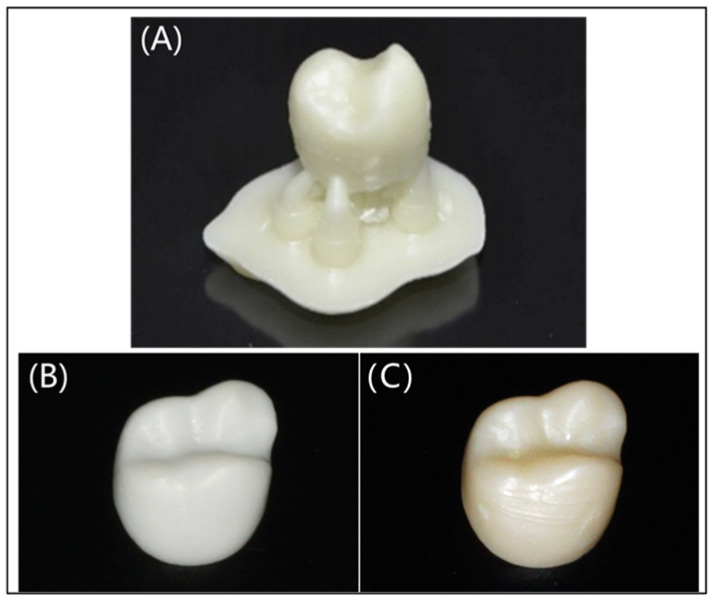
Three-dimensional printed crown. (**A**) Example of 3D printed crown before polishing and removal of supports. (**B**) The 3D printed crown restoration. (**C**) The subtractive manufactured crown restoration. Reprinted with permission from Elsevier [33,34].

**Figure 6:**
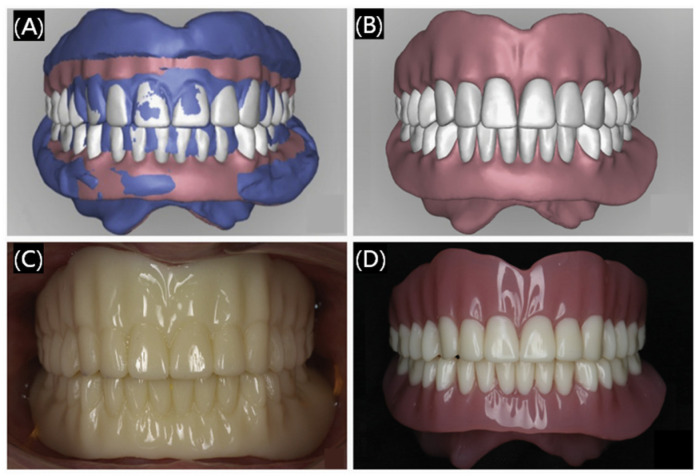
Three-dimensional printed dentures. (**A**) Superimposed image of 3D printed replication dentures and preview design. (**B**) Preview design frontal view. (**C**) Biofunctional evaluation of dentures frontal view. (**D**) Definitive digital dentures frontal view. Reprinted with permission from Elsevier [35].

Currently, 3D printing technology has been used for the production of customized removable partial dentures that are applied to restore damaged teeth, including crowns and bridges, dentitions, dental arches, and so on. The printing materials to make restorations are mainly resin and paraffin. Three-dimensional printed restorations are smoother and can better meet people’s esthetic requirements (Figure 5), helping patients to overcome the pain caused by product mismatches and reduce discomfort and long-term residual bone resorption [33,34]. Not limited to printing the local structure of the teeth, at the same time, researchers have been working on printing the whole denture. 3D printed dentures are also very popular, as shown in Figure 6 [35], some preparation processes of 3D printing dentures can be personalized and customized to better match the different oral conditions of different people. Furthermore, 3D printing technology will greatly save the materials reduce the costs and shorten the waiting time of the patients. Overall, 3D printing dental restorations are very attractive and will be more and more commonly applied in dental prosthetics, which is very promising for their widespread spread in clinical applications [36].

#### 3.1.4. Dental Orthodontics

**Figure 7:**
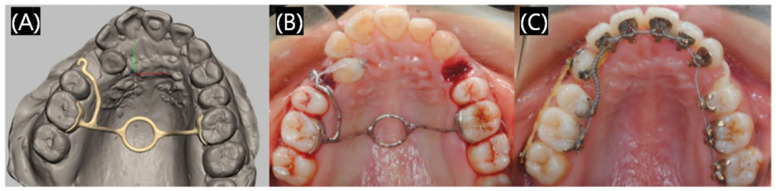
A case of 3D printed trans-palatal arches applied in the orthodontic field. (**A**) The clinician designed 3D trans-palatal arche with a lateral hook to pull the canine (**B**) The 3D printed trans-palatal arche was applied intraorally. (**C**) The malposed canine was corrected successfully with the 3D printed trans-palatal arche. Reprinted with permission from MDPI [38].

Orthodontics is a branch of dentistry that deals with the diagnosis and correction of malpositioned teeth and jaws [37], which can help patients restore beauty, health, and confidence, and orthodontics is always a field of significant interest in dentistry. During the process of orthodontia, using medical devices like models, aligners, and retainers is essential. The traditional method to prepare these models or devices is hot-pressing, and sometimes it is difficult to control the exact structure, with satisfying the dynamic process during the orthodontia being especially impossible. Because 3D digital technology can predict the possible movement of teeth and design personalized, multi-functional products, 3D printed orthodontics has gradually prevailed in the market. Since the invention of 3D printing orthodontics, 3D printing technology can be customized to meet the accuracy, comfort, and personalized requirements of orthodontic treatment. As shown in Figure 7, in order to correct a poorly positioned canine tooth, the clinician first obtained a digital tooth model by scanning the plaster cast. Then, a special customized 3D printed trans-palatal arche with side hooks were designed to tow the canines (Figure 7A,B). Finally, the poorly positioned canines were corrected, the lingual brackets were bonded, and the lingual arch wires were successfully applied (Figure 7C) [38].

**Figure 8:**
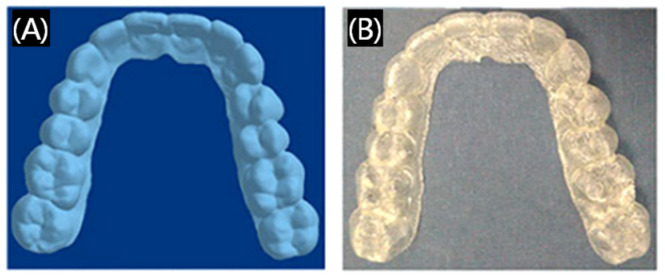
(**A**) 3D CAD drawing of an orthodontic aligner; (**B**) the aligner fabricated by 3D printing. Reprinted with permission from John Wiley and Sons [39].

Although 3D printing invisible appliances have the advantages of smooth outer surface and fewer local accessories compared with traditional dental appliances, there are still problems of poor durability or biocompatibility. Therefore, there is lot of research devoted to addressing these problems, for example, a modified hydroxyapatite occlusal composite with improved mechanical properties and antimicrobial capability was developed, increasing the adhesion of the filler with the polymer matrix (Figure 8) [39]. Therefore, the research and development of 3D printing materials of orthodontic devices with excellent biocompatibility, which can be stable, safe, and applied long-term in an oral environment, is still the most popular topic in current research.

#### 3.1.5. Others

**Figure 9:**
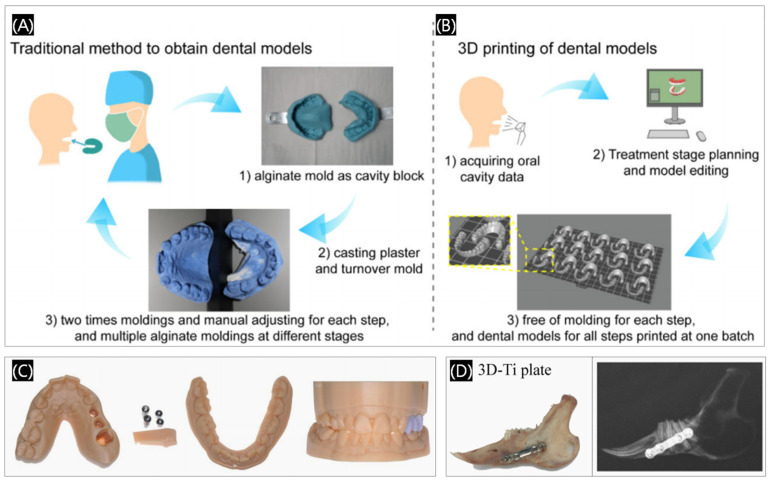
Comparison between (**A**) the traditional plaster casting method and (**B**) the 3D printing technique to obtain dental models. Reprinted with permission from Oxford University Press [40]. (**C**) Three-dimensional printed tooth models for implantology. (**D**) Three-dimensional printed Ti alloy plates. Reprinted with permission from MDPI [41,42].

In addition to the common application of 3D printing in dentistry mentioned above, 3D printing technology has currently been commonly applied in the field of surgical fixation plate, dental model making, dental education, and root canal surgery (Figure 9) [40,41,42].

To further support the above, in dentistry, 3D printing technology has been widely used in the experimental, educational, commercial, and clinical fields in recent decades [43]. Soon, 3D printing technology is expected to penetrate into all aspects of the field of stomatology, from clinical surgery to practical teaching to scientific research experiments.

### 3.2. The Application of 3D Printing Technology in Oral Tissue Regeneration

Dental tissue defects, often caused by severe diseases or trauma, pose significant challenges in clinical treatment due to their limited regenerative capacity [44,45]. This involves the study of tissue engineering, which is an interdisciplinary field combining biology and engineering principles, and offers a promising approach to developing functional substitutes for damaged tissues [46,47]. Three key elements are central to this approach: cells, scaffolds, and growth factors. In particular, scaffolds play a critical role by providing structural support for cell growth, proliferation, and tissue repair [48]. A wide range of materials has been investigated for scaffold fabrication, including inorganic materials (e.g., metal alloys, bioceramics), polymer materials (e.g., natural and synthetic polymers), and composite materials (e.g., nanoparticle/polymer blends). Additionally, the choice of manufacturing technology is crucial, with methods such as electrospinning [49], phase separation, and 3D printing being widely utilized. Among these, 3D printing stands out for its ability to precisely control scaffold shape and size. Three-dimensional printing technology enables precise and personalized scaffold fabrication, utilizing computer-aided design to create structures with defined shapes and internal architectures, making it a promising solution for oral tissue engineering.

The application of 3D printing in tissue regeneration was first proposed 20 years ago by Mironov et al. [50]. Three-dimensional printing of tissue engineering scaffolds not only emerged in oral therapy, but was already applied to repair and replace all kinds of diseased tissues and organs, for example, bone tissue regeneration [51], cartilage regeneration [52], nerve regeneration [53,54], vascular regeneration [55], and skin regeneration [56,57]. More critically, 3D printing scaffolds can mimic the hierarchical structure of native bone, providing a physiological micro-environment and transmitting growth factors, helping to regulate the migration, proliferation, differentiation, and extracellular matrix production of stem cells, which is beneficial for the regeneration of dental tissue. Therefore, 3D printing of oral tissue engineering scaffold is a very popular research topic. Next, according to the classification of different materials, several common 3D printed scaffolds in oral tissue engineering are introduced.

#### 3.2.1. Three-Dimensional Printed Metal-Based Scaffolds

**Figure 10:**
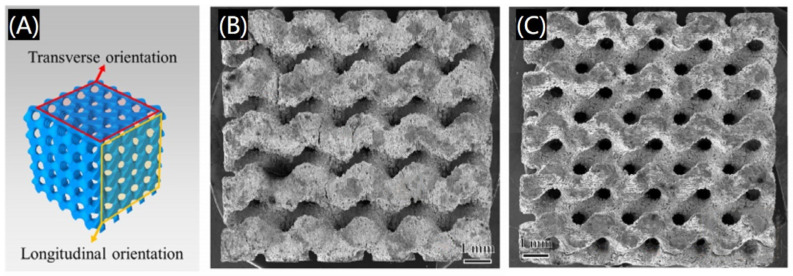
Three-dimensional printing of Zn-Mg alloy scaffold. (**A**) Schematic diagram of the transverse and longitudinal orientations. (**B**) Longitudinal orientation view and (**C**) transverse orientation view. Reprinted with permission from Elsevier [58].

Metals and metal alloys are original and commonly used raw materials in the clinical use of dentistry, and with the rise of 3D printing technology, 3D printed metal-based scaffolds have also come into being, metal material for hard tissue repair of the oral and bone treatments such as titanium, magnesium, zinc, and various alloys. For example, additive manufactured biodegradable Zn alloy scaffolds constitute an important branch in orthopedic implants because of their moderate degradation behavior and bone-mimicking mechanical properties. A 3D printed Zn-Mg scaffold with high fatigue strength and fatigue resistance was prepared (Figure 10) [58], which can promote the application of Zn-Mg scaffolds in the treatment of bone defects.

Due to their cost-effectiveness and ease of processing, metal-based materials have remained a focal point of research in the development of 3D printed scaffolds for clinical dental applications. However, the inherent inertness of these materials poses a significant limitation, prompting ongoing investigations into strategies for enhancing their bioactivity, such as surface functionalization or bioactive coating design. More advancements aim to enable these scaffolds to more effectively support true tissue regeneration within the human body, thereby improving their clinical utility and functional integration.

#### 3.2.2. Three-Dimensional Printed Ceramic-Based Scaffolds

Bioactive materials are considered excellent alternatives to hard tissue, particularly bone tissue and related topics in bone tissue engineering and dental restoration [59]. In recent years, new ceramic materials with good biological activity have also come to the fore. Bioceramic is an inorganic biomaterial used in bone tissue engineering scaffolds, mainly including calcium phosphate, bone cement, bioactive glass, alumina, and zirconia, all of which have excellent bone conductivity. Therefore, numerous studies on 3D printing bioceramic materials have emerged.

**Figure 11:**
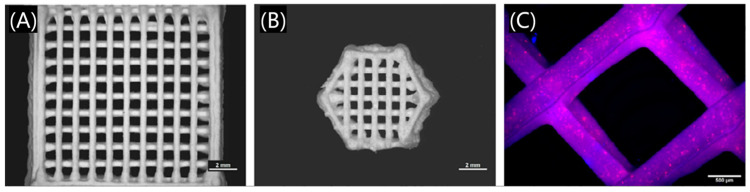
Composites consisting of calcium phosphate cements and mesoporous bioactive glass scaffolds consisting of four layers with (**A**) a 90° layer-to-layer orientation and (**B**) a hexagonal geometry. (**C**) Fluorescence image of a 3D plotted scaffold. Reprinted with permission from Elsevier [61].

For example, calcium phosphate cement (CPC) and mesoporous bioactive glass (MBG) are two widely studied biomaterials whose applicability to bone defects in orthopedic and maxillofacial surgery have been extensively studied. Richter et al. have been working on the creation of composite raw materials comprising CPC and MBG, two promising regenerative and degradable biomaterials, to make 3D printable scaffolds for drug delivery systems (Figure 11) that can create implants with patient-specific geometries [60]. Pan et al. prepared a 3D printed dual-response borosilicate glass (BSG) scaffold for alveolar bone defect repair, which is expected to be applied to alveolar bone damage caused by diabetes [61].

**Figure 12:**
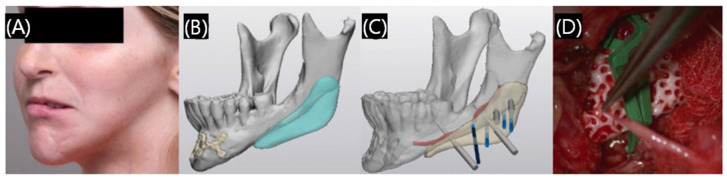
Planning and surgery of 3D printed implant with the treatment of craniomaxillofacial defect. (**A**) Preoperative clinical conditions. (**B**) Digital design of the scaffold and (**C**) the digital design of fixing screws related to the alveolar nerve. (**D**) Placement and fixation of scaffold during surgery. Reprinted with permission from MDPI [62].

In addition to theoretical research, 3D printed ceramic-based scaffolds have been used in clinical treatment. As shown in Figure 12 [62], a 19-year-old female patient with a craniomaxillofacial bone defect was treated with a patient-specific 3D printed hydroxyapatite bioceramic implant. Satisfactory esthetic results were found in this clinical case, and the implant was stable with no bone resorption or infection. Future research will also focus on the microstructures and multiple composite materials through physical or chemical modifications, as well as giving biological functionalization to them, making it truly more extended to clinical practice.

#### 3.2.3. Three-Dimensional Printed Polymer-Based Scaffolds

Polymer scaffolds, particularly degradable ones, have garnered significant attention in recent years within the field of biological tissue engineering [63]. These polymers are broadly categorized into natural and synthetic types. Natural polymers, such as gelatin, collagen, chitosan, alginic acid, and hyaluronic acid, are widely used due to their biocompatibility and biodegradability. In contrast, synthetic polymers, including polylactic acid (PLA), polycaprolactone (PCL), and polylactic acid–polyglycolic acid copolymer (PLGA), offer superior mechanical strength alongside biodegradability, making them ideal for 3D printed scaffolds in oral and bone regeneration. Their enhanced structural properties position synthetic polymers as excellent candidates for addressing complex bone defects.

**Figure 13:**
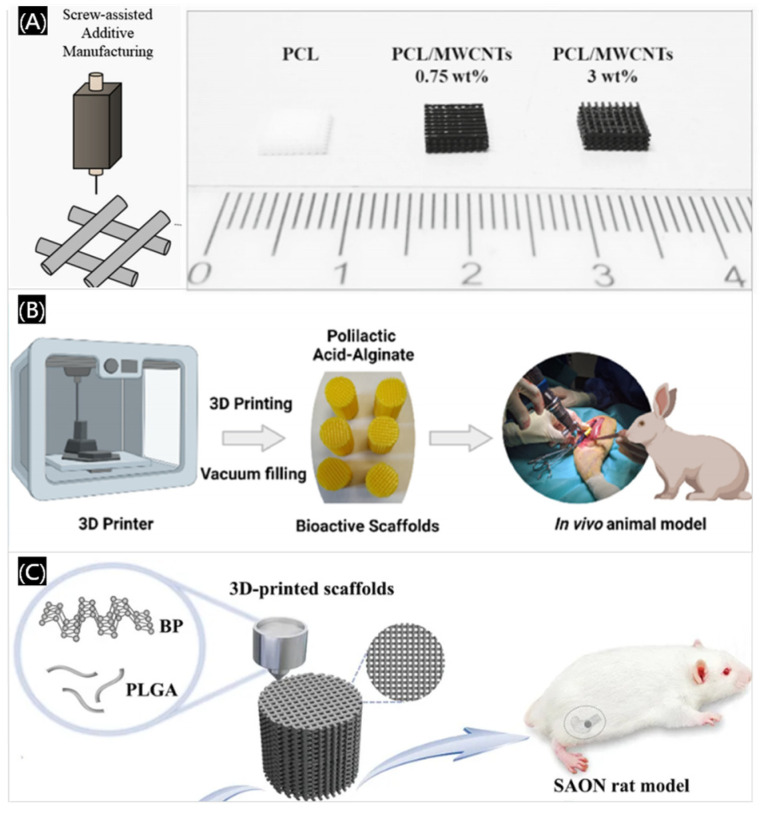
Three-dimensional printed polymer-based scaffolds. (**A**) Three-dimensional printed PCL-based implanted scaffolds. Reprinted with permission [64]. (**B**) Three-dimensional printed PLA and alginate polymers composite scaffolds. Reprinted with permission from American Chemical Society [65]. (**C**) PLGA/BP scaffolds fabricated by 3D printing. Reprinted with permission from John Wiley and Sons [69].

Recent advancements in 3D printing have demonstrated the potential of synthetic polymer scaffolds in bone regeneration. For instance, conductive scaffolds composed of PCL and multi-walled carbon nanotubes (MWCNTs) were fabricated using extrusion-based additive manufacturing, effectively treating large calvarial bone defects in rats (Figure 13A) [64]. Similarly, composite 3D printed scaffolds combining PLA and alginate, developed by Sanjukta Deb’s team, exhibited antimicrobial and osteoinductive properties, promoting bone regeneration in rabbit models (Figure 13B) [65]. In addition, there are also bone defects in other parts of the body that require precise technology to repair, so 3D printing is suitable for many bone repair fields, just like the clinically challenging critical bone defects [66,67], such as incorporating nano ZIF-8 into PCL and dicalcium phosphate dihydrate scaffolds, which has shown significant improvements in calvarial defect repair in rabbits [68]. Additionally, PLGA/black phosphorus (BP) scaffolds have been shown to modulate the bone immune microenvironment and enhance osteogenesis, particularly in steroid-associated osteonecrosis models (Figure 13C) [69]. Therefore, 3D printed polymer-based scaffolds are promising in treating bone defects that are difficult to heal.

Despite these advancements, the biocompatibility and long-term safety of 3D printed scaffolds remain a challenge. Implanted materials can trigger inflammatory responses, particularly through macrophage-mediated chronic inflammation, which may compromise regeneration and lead to implant failure [70,71]. Therefore, the development of bioactive composite scaffolds with immunomodulatory properties is a key research focus [72].

#### 3.2.4. Three-Dimensional Printed Composite Bioactive Scaffolds

3D printed composite bioactive scaffolds are fabricated by combining diverse materials to optimize design and functionality. First, materials such as polymers, metals, or ceramics, are selected based on specific application requirements, while reinforcing agents like bioactive glass, carbon fibers, or nanoparticles are incorporated to enhance mechanical strength and biocompatibility [73]. These composite scaffolds are particularly effective in delivery systems, where their integration with growth factors or therapeutic drugs demonstrates significant potential in regenerative medicine. The controlled release of such bioactive agents is critical for promoting angiogenesis and osteogenesis [74].

**Figure 14:**
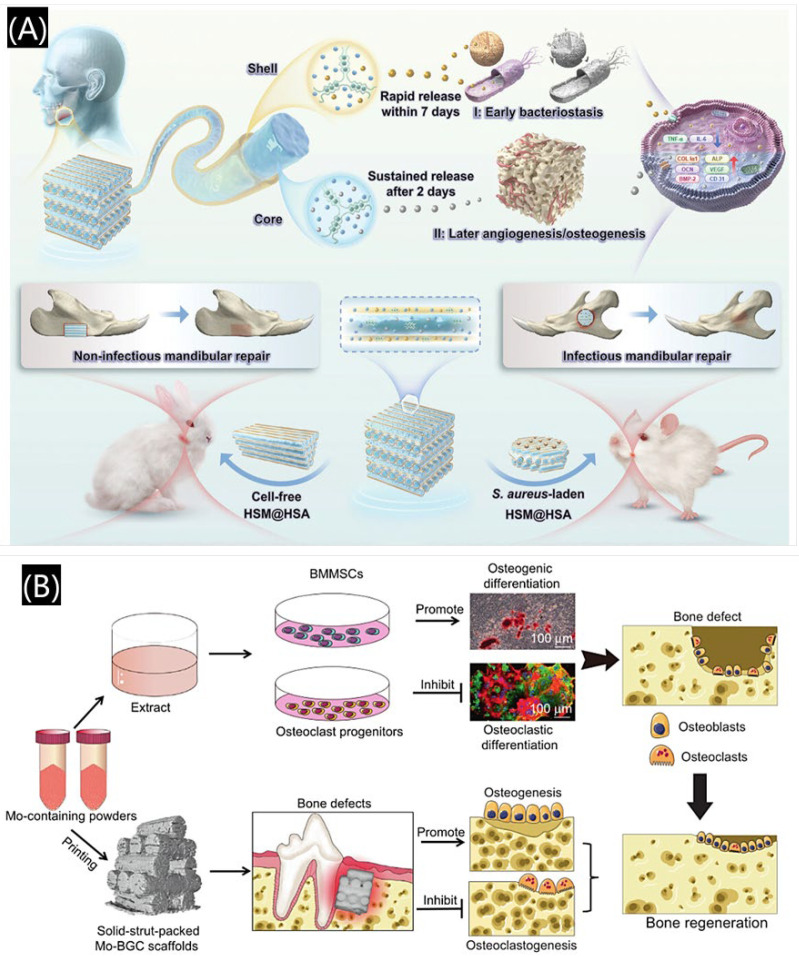
Three-dimensional printed composite bioactive scaffolds. (**A**) A 3D printed composite scaffold loaded with antibiotics and Chinese medicine molecules. Reprinted with permission from John Wiley and Sons [75]. (**B**) 3D printing molybdenum-loaded immunomodulatory MBG scaffold. Reprinted with permission from Elsevier [78].

To date, numerous 3D printed composite bioactive scaffolds incorporating biological factors or drugs have been developed, highlighting their potential for clinical applications in tissue regeneration and repair. For example, a coaxial 3D printed scaffold was developed, which consisted of minocycline hydrochloride and deer horn powder demonstrated antimicrobial and osteogenic capabilities, effectively repairing infected mandibular defects (Figure 14A) [75]. In addition to biological factors or drugs, ions also show strong osteogenic potential, making them ideal for designing bioactive regenerative materials [76,77]. For instance, He et al. developed molybdenum-containing bioactive glass–ceramic (Mo-BGC) scaffolds using 3D printing, which showed immunomodulatory properties and facilitated alveolar bone restoration. Molybdenum, as a functional loading element, has immunomodulatory effects, which can regulate local immune responses and promote tissue repair (Figure 14B) [78]. Ensuring immune compatibility and minimizing inflammatory responses are essential for the successful clinical translation of 3D printed scaffolds. Therefore, the research and development of biologically active composite scaffolds, especially those with immunomodulatory effects, has been a topic worthy of attention in recent years.

#### 3.2.5. Three-Dimensional Bioprinted Scaffolds

**Figure 15:**
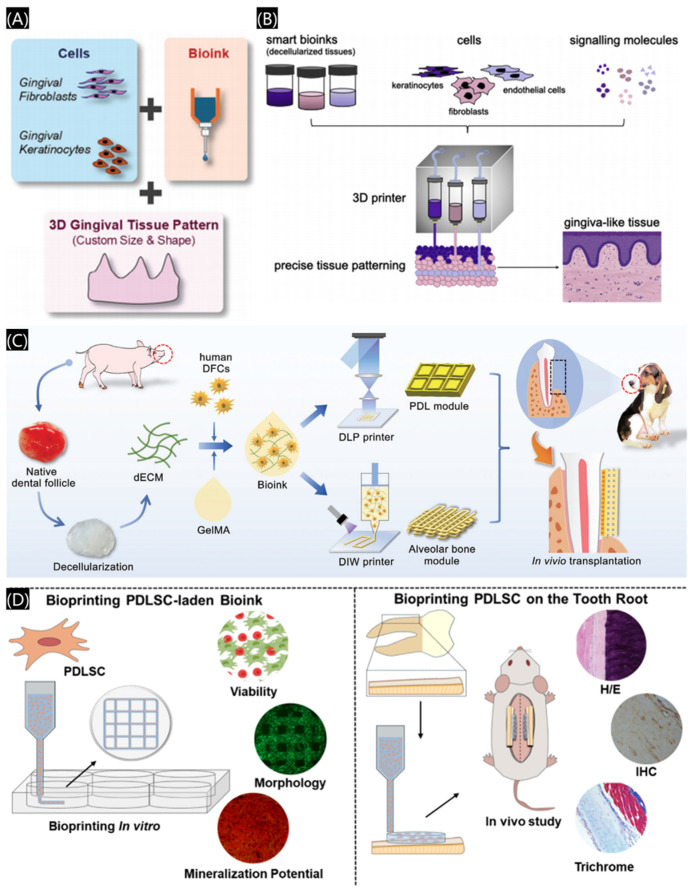
Three-dimensional bioprinting applications in the field of dentistry. (**A**) Biocompatible bioink laden with cells. Reprinted with permission from John Wiley and Sons [79]. (**B**) A combination of smart bioinks for the fabrication of a more nature-like gingiva tissue. Reprinted with permission from Elsevier [85]. (**C**) Three-dimensional bioprinting using GelMA/dECM cell-laden bioink to restore periodontal hard and soft tissues. Reprinted with permission from Elsevier [86]. (**D**) Scaffolds with collagen and PDLSC-laden bioinks for periodontal ligament regeneration. Reprinted with permission from the American Chemical Society [87].

3D bioprinting, an advanced extension of 3D printing, fabricates cell-laden structures with high precision by depositing bioinks composed of biomaterials, cells, and biological factors in a layer-by-layer method. (Figure 15A) [79]. Three-dimensional bioprinting consists of four key elements: bioink formulation, model structure, the printing process, and function regulation [80].

The bioink is critical in bioprinting [81], and various materials have been widely used as bioinks, including synthetic and natural polymers. For example, collagen, a natural polymer, which is widely distributed in skin, bone, and other tissues, have been broadly used in 3D bioprinting [82]. Gelatin, a hydrophilic colloid from partially degraded collagen, often combines with other materials, for example, gelatin methacrylic acid (GelMA), which is also a kind of common material used for bioinks. In addition, other polymers, such as hyaluronic acid methacrylic-modified hyaluronic acid (HAMA) and sodium alginate are all excellent candidates for bioinks of 3D bioprinting. In advanced bioprinting technologies, the control of bioink composition and mechanical properties is critical. Therefore, in order to adapt the properties of bioinks to specific applications, materials of natural origin can be combined with other polymer materials to form composites. For example, polyethylene glycol (PEG) is often used to prepare 3D printing bioinks to improve the stability of printed structures and cell viability [83]. The addition of biodegradable polymers, such as polylactic acid–polyglycolic acid (PLGA), can enhance the structural stability of the printed structure [84]. Therefore, natural polymers are combined with polymer materials to form composite materials, which can better meet specific bioprinting needs.

3D bioprinting offers innovative solutions for periodontal tissue regeneration, bone defect repair, and tooth reconstruction. By stacking biocompatible materials and living cells, it accurately replicates the complex structures of oral tissues. For periodontal disease treatment, customized biological scaffolds can be fabricated to match patient-specific defects. In addition to hard tissues, 3D bioprinting has expanded its applications to include the regeneration of soft tissues, particularly gingival and mucosal structures. Bioinks enable the co-printing of cells in specific architectural designs, creating natural-looking gingival-like tissues (Figure 15B) [85]. For instance, a 3D bioprinted biomimetic periodontal module is designed with high architectural integrity using a methacrylate gelatin/decellularized extracellular matrix (GelMA/dECM) cell-laden bioink. (Figure 15C) [86]. As shown in Figure 15D, a collagen-based bioink mimicking the native extracellular matrix conditions and carrying periodontal ligament stem cells (PDLSCs) was tested to guide the periodontal ligament organization [87]. These advancements, combined with stem cell technology and biomimetic scaffolds, hold significant promise for enhancing dental tissue regeneration [88].

In summary, 3D bioprinting represents a cutting-edge integration of biomaterials science, cell biology, and precision manufacturing [89]. It not only enables the fabrication of hard and soft oral tissues but also provides superior control over cell and biological factor distribution within scaffolds, making it highly suitable for the complex oral physiological environment [90].

## 4. Conclusions and Perspectives

In conclusion, 3D printing technology, as a simple and effective manufacturing method, has been widely used in dental medicine, some commercial medical products have been used in clinics. Although 3D printing technology has attracted great attention in dentistry, it still faces many challenges to pave the way of application in clinic. Firstly, the materials that can satisfy the clinic application are still limited, for example, materials with proper mechanical properties that can be superior to traditional ceramics or metal alloys are still needed [91]. Meanwhile, the costs of materials and equipment are still too high to be widespread; in addition, the post-processing procedure are complex. Furthermore, the clinical test is indeed a time-consuming and effort-intensive process, and thus, it is a long way for the 3D printing product to go from the laboratory to the clinic. Therefore, addressing these limitations is crucial for the advancement of 3D printed dental materials. In addition, dental tissue regeneration engineering is still not systematic and mature, and its clinical application is relatively limited. Three-dimensional printing is also a popular topic in tissue engineering or regenerative medicine to construct scaffolds with sophisticated structures. Therefore, 3D printing is a perspective technology in modern medicine.

In the next few years, 3D printing will be combined together with biology, chemistry, or life science, and scaffolds with bioactive factors or living cells will be widely investigated. Furthermore, four-dimensional (4D) printing, which can add active and responsive functions to three-dimensional (3D) printed objects [92,93], represents a cutting-edge research direction, combining spatial and temporal control to create more sophisticated and functional scaffolds. In addition, designing new materials, reducing the cost of materials and equipment, and accelerating the translation from laboratory to clinic are inevitable to promote the application of 3D printing in dentistry.

With the rapid progress of technology, we are sure that 3D printing technology will fully meet the needs of clinical applications and bring significant benefits to patients. However, only by continuously integrating dental medicine with other disciplines (biology, chemistry, computer science, information technology) can the true potential of this technology be realized in both scientific research and clinical environments. More importantly, researchers, clinicians, and regulatory bodies should cooperate together; only through this, 3D printing from the laboratory to clinical applications can be greatly improved and ultimately promote the advancement of dentistry.

## Figures and Tables

**Table 1:** The raw materials, advantages, challenges and costs of different 3D printing.

Techniques	Raw Materials	Advantages	Challenges	Costs	Refs.
Selective laser melting	Metal powders: stainless steel powder, iron powder, etc.	High precision, efficiency and complex structure manufacturing	Limited materials and time-consuming	The manufacturing cost is higher	[12,13,14,15]
Selective laser sintering	Polymers, metals, ceramics, gypsum, and their mixed powders	Wide range of material adaptability, suitable for mass production	Slow printing speeds and complex post-processing	The cost of equipment is higher	[16]
Fused deposition modeling	Filamentous thermoplastic materials: PLA, nylon, etc.	High efficiency and high material utilization	Slow printing speed and limited accuracy	Economical and practical	[17]
Stereolithography	Liquid photopolymer resin	High precision, smooth printing surface	Time-consuming and complex post-processing	The cost of materials, equipment and its maintenance are higher	[18,19]
Digital light processing	Photopolymer liquid and ceramic mixture slurry	High precision, high efficiency and fast printing speed	Limited print size and operating temperature	Cost-effective	[20,21,22]

## Data Availability

The data presented in this study are available on request from the corresponding author.

## References

[1] Laleh M., Sadeghi E., Revilla R.I., Chao Q., Haghdadi N., Hughes A.E., Xu W., Graeve I.D., Qian M., Gibson I. (2023). Heat treatment for metal additive manufacturing. Prog. Mater. Sci..

[2] Ligon S.C., Liska R., Stampfl J., Gurr M., Mülhaupt R. (2017). Polymers for 3D printing and customized additive manufacturing. Chem. Rev..

[3] Quan H.Y., Zhang T., Xu H., Luo S., Nie J., Zhu X.Q. (2020). Photo-curing 3D printing technique and its challenges. Bioact. Mater..

[4] Afshar A., Mihut D. (2020). Enhancing durability of 3D printed polymer structures by metallization. J. Mater. Sci. Technol..

[5] Liu G., He Y.H., Liu P.C., Chen Z., Chen X.L., Wan L., Li Y., Lu J. (2020). Development of bioimplants with 2D, 3D, and 4D additive manufacturing materials. Engineering.

[6] Ghai S., Sharma Y., Jain N., Satpathy M., Pillai A.K. (2018). Use of 3-D printing technologies in craniomaxillofacial surgery: A review. Oral Maxillofac. Surg..

[7] Yu J., Bian H.L., Zhao Y.N., Guo J.M., Yao C.M., Liu H., Shen Y., Yang H.Y., Huang C. (2023). Epigallocatechin-3-gallate/mineralization precursors co-delivery hollow mesoporous nanosystem for synergistic manipulation of dentin exposure. Bioact. Mater..

[8] Tigmeanu C.V., Ardelean L.C., Rusu L.C., Negrutiu M.L. (2022). Additive Manufactured Polymers in Dentistry, Current State-of-the-Art and Future Perspectives-A Review. Polymers.

[9] Meng L., Bai J.X., Tao H.Q., Hao L., Yin W.L., Ren X.X., Gao A., Li N., Wang M., Fang S.Y. (2022). Rational integration of defense and repair synergy on PEEK osteoimplants via biomimetic peptide clicking strategy. Bioact. Mater..

[10] Lin L.W., Fang Y.F., Liao Y.X., Chen G., Gao C.X., Zhu P.Z. (2019). 3D printing and digital processing techniques in dentistry: A review of literature. Adv. Eng. Mater..

[11] Liu X.M., Zhao D., Wang J. (2024). Challenges and opportunities in preserving key structural features of 3D-printed metal/covalent organic framework. Nano-Micro Lett..

[12] Wang C.Z., Hu Y.X., Zhong C., Lan C.X., Li W., Wang X.J. (2022). Microstructural evolution and mechanical properties of pure Zn fabricated by selective laser melting. Mat. Sci. Eng. A.

[13] Kouhi M., Araújo I.J.S., Asa’ad F., Zeenat L., Bojedla S.S.R., Pati F. (2024). Recent advances in additive manufacturing of patient-specific devices for dental and maxillofacial rehabilitation. Dental Mats..

[14] Takahashi A., Inoue K., Imagawa-Fujimura N., Matsumoto K., Yamada K., Sawai Y., Nakajima Y., Mano T., Kato-Kogoe N., Ueno T. (2023). Clinical study of 14 cases of bone augmentation with selective laser melting titanium mesh plates. Materials.

[15] Park J.H., Odkhuu M., Cho S., Li J.W., Park B.-Y., Kim J.-W. (2020). 3D-printed titanium implant with premounted dental implants for mandible reconstruction: A case report. Maxillofac. Plast. Reconstr. Surg..

[16] Zhang F., Zhou S.X., You H.Y., Zhang G., Yang J.Q., Shi Y.S. (2025). 3D printing of ceramic matrix composites: Strengthening and toughening strategies. Compos. Part B.

[17] Ahn S.J., Lee H., Cho K.J. (2024). 3D printing with a 3D printed digital material filament for programming functional gradients. Nat. Commun..

[18] Bagheri A., Jin J.Y. (2019). Photopolymerization in 3D Printing. ACS Appl. Polym. Mater..

[19] Zhou X., Yu X., You T., Zhao B., Dong L., Huang C., Zhou X., Qian W., Luo G. (2024). 3D Printing-Based Hydrogel Dressings for Wound Healing. Adv. Sci..

[20] González G., Baruffaldi D., Martinengo C., Angelini A., Chiappone A., Roppolo I., Pirri C.F., Frascella F. (2020). Materials testing for the development of biocompatible devices through vat-polymerization 3D printing. Nanomaterials.

[21] Dhand A.P., Davidson M.D., Burdick J.A. (2025). Lithography-based 3D printing of hydrogels. Nat. Rev. Bioeng..

[22] Wan X., Xiao Z.M., Tian Y.J., Chen M., Liu F., Wang D. (2024). Recent advances in 4D printing of advanced materials and structures for functional applications. Adv. Mater..

[23] Song C.H., Liu R., Kong B., Gu Z.X., Chen G.P. (2024). Functional hydrogels for treatment of dental caries. Biomed. Technol..

[24] Tarce M., Joe Merheb J., Meeus M., Vasconcelos K.D.F., Marc Quirynen M. (2022). Surgical guides for guided bone augmentation: An in vitro study. Clin. Oral. Impl. Res..

[25] Tian Y., Chen C.X., Xu X.T., Wang J.Y., Hou X.Y., Li K.L., Lu X.Y., Shi H.Y., Lee E.S., Jian H.B. (2021). A Review of 3D Printing in Dentistry: Technologies, Affecting Factors, and Applications. Scanning.

[26] Xu L.W., You J., Zhang J.X., Liu Y.F., Peng W. (2016). Impact of surgical template on the accuracy of implant placement. J. Prosthodont..

[27] Kholy K.E., Lazarin R., Janner S.F.M., Faerber K., Buser R., Buser D. (2019). Influence of surgical guide support and implant site location on accuracy of static Computer-Assisted Implant Surgery. Clin. Oral Implants Res..

[28] Vasoglou G., Patatou A., Vasoglou M. (2023). Bimaxillary dentoalveolar protrusion case treated with anchorage by buccally implemented mini-implants using a 3D-printed surgical guide. Children.

[29] Sonaye S.Y., Bokam V.K., Saini A., Nayak V.V., Witek LCoelho P.G., Bhaduri S.B., Bottino M.C., Sikder P. (2022). Patient-specific 3D printed Poly-ether-ether-ketone (PEEK) dental implant system. J. Mech. Behav. Biomed. Mater..

[30] Turkyilmaz I., Wilkins G.N. (2021). 3D printing in dentistry—Exploring the new horizons. J. Dent. Sci..

[31] Brink T., Damanik F., Rotmans J., Moroni L. (2024). Unraveling and harnessing the immune response at the cell–biomaterial interface for tissue engineering purposes. Adv. Healthcare Mater..

[32] Makvandi P., Gu J.T., Zare E.N., Ashtari B., Moeini A., Tay F.R., Niu L.N. (2020). Polymeric and inorganic nanoscopical antimicrobial fillers in dentistry. Acta Biomater..

[33] Tahayeri A., Morgana M.C., Fugolin A.P., Bompolaki D., Athirasala A., Pfeifer C.S., Ferracane J.L., Bertassoni L.E. (2018). 3D printed versus conventionally cured provisional crown and bridge dental materials. Dent. Mater..

[34] Wang W.N., Yu H., Liu Y.F., Jiang X.L., Ga B. (2019). Trueness analysis of zirconia crowns fabricated with 3-dimensional printing. J. Prosthet. Dent..

[35] Takeda Y., Lau J., Nouh H., Hirayama H. (2020). A 3D printing replication technique for fabricating digital dentures. J. Prosthet. Dent..

[36] Oberoi G., Nitsch S., Edelmayer M., Janjić K., Müller A.S., Agis H. (2018). 3D printing-encompassing the facets of dentistry. Front. Bioeng. Biotech..

[37] Schweiger J., Edelhoff D., Güth J.F. (2021). 3D printing in digital prosthetic dentistry: An overview of recent developments in additive manufacturing. J. Clin. Med..

[38] Kuang Y., Hu B., Feng G., Huang L., Song J. (2022). The Application of a 3-dimensional printing technique in refining the orthodontic trans-palatal. Arch. Appl. Sci..

[39] Makvandi P., Esposito Corcione C.E., Paladini F., Gallo A.L., Montagna F., Jamaledin R., Pollini M., Maffezzoli A. (2018). Antimicrobial modified hydroxyapatite composite dental bite by stereolithography. Poly. Advan. Technol..

[40] Yu X.Y., Li G.H., Zheng Y.K., Pan X.G., Ding J.D. (2022). ‘Invisible’ orthodontics by polymeric ‘clear’ aligners molded on 3D-printed personalized dental models. Regen. Biomater..

[41] Wang QTelha W., Wu Y., Abotaleb B., Jiang N., Zhu S. (2023). Evaluation of the properties of 3D-printed Ti alloy plates: In Vivo and In Vitro Comparative Experimental Study. J. Clin. Med..

[42] Zhang Q., Wu W., Qian C.Y., Xiao W.S., Zhu H.J., Guo J., Meng Z.B., Zhu J.Y., Ge Z.L., Cui W.G. (2019). Advanced biomaterials for repairing and reconstruction of mandibular defects. Mater. Sci. Eng. C.

[43] Guo J.X., Yao H., Li X., Chang L., Zhu W.Y., Su Y.X., Qin L., Xu J.K. (2023). Advanced hydrogel systems for mandibular reconstruction. Bioact. Mater..

[44] Zhao F.J., Yang Z., Xiong H.C., Yan Y., Chen X.F., Shao L.Q. (2023). A bioactive glass functional hydrogel enhances bone augmentation via synergistic angiogenesis, self-swelling and osteogenesis. Bioact. Mater..

[45] Cidonioa G., Glinkaa M., Dawsona J.I., Oreffo R.O.C. (2019). The cell in the ink: Improving biofabrication by printing stem cells for skeletal regenerative medicine. Biomaterials.

[46] Langer R., Vacanti J.P. (1993). Tissue Engineering. Science.

[47] Masri S., Zawani M., Zulkiflee I., Salleh A., Fadilah N.I.M., Maarof M., Wen A.P.Y., Duman F., Tabata Y., Aziz I.A. (2022). Cellular Interaction of Human Skin Cells towards Natural Bioink via 3D-Bioprinting Technologies for Chronic Wound: A Comprehensive Review. Int. J. Mol. Sci..

[48] Gao J.M., Yu X.Y., Wang X.L., He Y.N., Ding J.D. (2022). Biomaterial-related cell microenvironment in tissue engineering and regenerative medicine. Engineering.

[49] Yan N., Hu B., Xu J.C., Cai R., Liu Z.H., Fu D.P., Huo B.B., Liu Z.H., Zhao Y.L., Chen C.Y. (2022). Stem cell Janus patch for periodontal regeneration. Nano Today.

[50] Mironov V., Boland T., Trusk T., Forgacs G., Markwald R.R. (2003). Organ printing: Computer-aided jet-based 3D tissue engineering. Trends Biotechnol..

[51] Wang C., Huang W., Zhou Y., He L.B., He Z., Chen Z.L., He X., Tian S., Liao J.M., Lu B.H. (2020). 3D printing of bone tissue engineering scaffolds. Bioact. Mater..

[52] Li Q.T., Xu S., Feng Q., Dai Q.Y., Yao L.T., Zhang Y.C., Gao H.C., Dong H., Chen D.F., Cao X.D. (2021). 3D printed silk-gelatin hydrogel scaffold with different porous structure and cell seeding strategy for cartilage regeneration. Bioact. Mater..

[53] Liu Z.B., Wang C.J., Fang Y.C., Ko J., Chen L., Zhang T., Xiong Z., Zhang L., Sun W. (2023). 3D printed conductive multiscale nerve guidance conduit with hierarchical fibers for peripheral nerve regeneration. Adv. Sci..

[54] Gong H., Fei H.S., Xu Q.F., Gou M.L., Chen H.H. (2020). 3D-engineered GelMA conduit filled with ECM promotes regeneration of peripheral nerve. J. Biomed. Mater. Res. A.

[55] Kolesky D.B., Truby R.L., Gladman A.S., Busbee T.A., Homan K.A., Lewis J.A. (2014). 3D Bioprinting of vascularized, heterogeneous cell-laden tissue constructs. Adv. Mater..

[56] Daikuara L.Y., Chen X.F., Yue Z.L., Skropeta D., Wood F.M., Fear M.W., Wallace G.G. (2022). 3D bioprinting constructs to facilitate skin regeneration. Adv. Funct. Mater..

[57] Kim B.S., Kwon Y.W., Kong J.S., Park G.T., Gao G., Han W., Kim M.B., Lee H., Kim J.H., Cho D.W. (2018). 3D cell printing of in vitro stabilized skin model and in vivo pre-vascularized skin patch using tissue-specific extracellular matrix bioink: A step towards advanced skin tissue engineering. Biomaterials.

[58] Zhao D.L., Han C.J., Peng B., Cheng T., Fan J.X., Yang L., Chen L.L., Wei Q.S. (2022). Corrosion fatigue behavior and anti-fatigue mechanisms of an additively manufactured biodegradable zinc-magnesium gyroid scaffold. Acta Biomater..

[59] Boccaccini A.R., Höland W. (2016). Editorial: Inorganic Biomaterials. Front. Bioeng. Biotechnol..

[60] Richter R.F., Ahlfeld T., Gelinsky M., Lode A. (2023). Composites consisting of calcium phosphate cements and mesoporous bioactive glasses as a 3D plottable drug delivery system. Acta Biomater..

[61] Tian P.F., Zhao L.M., Kim J., Li X., Liu C.Y., Cui X., Liang T., Du Y.B., Chen X.H., Pan H.B. (2023). Dual stimulus responsive borosilicate glass (BSG) scaffolds promote diabetic alveolar bone defectsrepair by modulating macrophage phenotype. Bioact. Mater..

[62] Verbist M., Vandevelde A.-L., Geusens J., Sun Y., Shaheen E., Willaert R. (2024). Reconstruction of craniomaxillofacial bone defects with 3D-printed bioceramic implants: Scoping review and clinical case series. J. Clin. Med..

[63] Wang Y.P., Wang J.R., Gao R., Liu X., Feng Z.J., Zhang C.N., Huang P.S., Dong A.J., Kong D.L., Wang W.W. (2022). Biomimetic glycopeptide hydrogel coated PCL/nHA scaffold for enhanced cranial bone regeneration via macrophage M2 polarization-induced osteo-immunomodulation. Biomaterials.

[64] Silva E.P., Huang B.Y., Helaehil J.V., Nalesso P.R.L., Bagne L., Oliveira M.A., Albiazetti G.C.C., Aldalbahi A., El-Newehy M., Santamaria-Jr M. (2021). In vivo study of conductive 3D printed PCL/MWCNTs scaffolds with electrical stimulation for bone tissue engineering. Bio-Des. Manuf..

[65] Serra-Aguado C.I., Llorens-Gamez M., Vercet-Llopis M., Martinez-Chicote V., Deb S., Serrano-Aroca A. (2022). Engineering Three-dimensional-printed bioactive polylactic acid alginate composite scaffolds with antibacterial and in vivo osteoinductive capacity. ACS Appl. Mater. Inter..

[66] Ma L.M., Wang X.L., Zhao N.R., Zhu Y., Qiu Z.Y., Li Q.T., Zhou Y., Lin Z.F., Li X., Zeng X.L. (2018). Integrating 3D printing and biomimetic mineralization for personalized enhanced osteogenesis, angiogenesis, and osteointegration. ACS Appl. Mater. Inter..

[67] Wang Y., Liu Y., Chen S.S., Siu M.F.F., Liu C., Bai J.M., Wang M. (2025). Enhancing bone regeneration through 3D printed biphasic calcium phosphate scaffolds featuring graded pore sizes. Bioact. Mater..

[68] Zhong L.N., Chen J.Y., Ma Z.Y., Feng H., Chen S., Cai H., Xue Y.Y., Pei X.B., Wang J., Wan Q.B. (2020). 3D printing of metal–organic framework incorporated porous scaffolds to promote osteogenic differentiation and bone regeneration. Nanoscale.

[69] Long J., Yao Z.Y., Zhang W., Liu B., Chen K.M., Li L., Teng B., Du X.F., Li C.R., Yu X.F. (2023). Regulation of osteoimmune microenvironment and osteogenesis by 3D-printed PLAG/black phosphorus scaffolds for bone regeneration. Adv. Sci..

[70] Lee J., Byun H., Madhurakkat Perikamana S.K., SLee S., Shin H. (2019). Current advances in immunomodulatory biomaterials for bone regeneration. Adv. Healthc. Mater..

[71] Niu Y.M., Wang Z.Z., Shi Y.C., Dong L., Wang C.M. (2021). Modulating macrophage activities to promote endogenous bone regeneration: Biological mechanisms and engineering approaches. Bioact. Mater..

[72] Wang Y.L., Zhang H., Hu Y., Jing Y.Y., Geng Z., Su J.C. (2022). Bone repair biomaterials: A perspective from immunomodulation. Adv. Funct. Mater..

[73] Wang J.W., Xia Y.H., Hao Z.W., Shi G., Zhang Q., Wang C.L., Zhu M.Y., Huang Y.L., Guo L.H., Luan T. (2025). A triple-integrated 3D-printed composite scaffold of high-activity peptide-metal ion-bone cement facilitates osteo-vascular regenerative repair of diabetic bone defects. Adv. Funct. Mater..

[74] Wu J.P., Liu Y.T., Cao Q.D., Yu T., Zhang J., Liu Q.Y., Yang X.Y. (2020). Growth factors enhanced angiogenesis and osteogenesis on polydopamine coated titanium surface for bone regeneration. Mater. Design.

[75] Zhang H., Sun H., Zhang L.L., Zhang B.Q., Zhang M., Luo Z.Y., Tan Y., Tang R., Sun J.X., Zhou X.D. (2024). Coaxial 3D printing scaffolds with sequential antibacterial and osteogenic functions to effectively repair infected mandibular defects. Adv. Funct. Mater..

[76] Su N., Villicana C., Yang F. (2022). Immunomodulatory strategies for bone regeneration: A review from the perspective of disease types. Biomaterials.

[77] Cui Y., Hong S.B., Jiang W.D., Li X.J., Zhou X.Y., He X.Y., Liu J.Q., Lin K.L., Mao L.X. (2024). Engineering mesoporous bioactive glasses for emerging stimuli-responsive drug delivery and theranostic applications. Bioact. Mater..

[78] He X.T., Li X., Zhang M., Tian B.M., Sun L.J., Bi C.S., Deng D.K., Zhou H., Qu H.L., Wu C.T. (2022). Role of molybdenum in material immunomodulation and periodontal wound healing: Targeting immunometabolism and mitochondrial function for macrophage modulation. Biomaterials.

[79] Dai Y.C., Wang P., Mishra A., You K., Zong Y.H., Lu W.F., Chow E.K.H., Preshaw P.M., Huang D.J., Chew J.R.J. (2024). 3D bioprinting and artificial intelligence-assisted biofabrication of personalized oral soft tissue constructs. Adv. Healthc. Mater..

[80] Zhang Z.R., Zhou X.H., Fang Y.C., Xiong Z., Zhang T. (2025). AI-driven 3D bioprinting for regenerative medicine: From bench to bedside. Bioact. Mater..

[81] Ostrovidov S., Salehi S., Costantini M., Suthiwanich K., Ebrahimi M., Sadeghian R.B., Fujie T., Shi X.T., Cannata S., Gargioli C. (2019). 3D bioprinting in skeletal muscle tissue engineering. Small.

[82] Martyniak K., Lokshina A., Cruz M.A., Karimzadeh M., Kemp R., JKean T.J. (2022). Biomaterial composition and stiffness as decisive properties of 3D bioprinted constructs for type II collagen stimulation. Acta Biomater..

[83] Rutz A.L., Gargus E.S., Hyland K.E., Lewis P.L., Setty A., Burghardt W.R., Shah R.N. (2019). Employing PEG crosslinkers to optimize cell viability in gel phase bioinks and tailor post printing mechanical properties. Acta Biomat..

[84] Choe G., Lee M., Oh S., Seok J.M., Kim J., Im S., Park S.A., Lee J.Y. (2022). Three-dimensional bioprinting of mesenchymal stem cells using an osteoinductive bioink containing alginate and BMP-2-loaded PLGA nanoparticles for bone tissue engineering. Biomater. Adv..

[85] Nesic D., Durual S., Marger L., Mekki M., Sailer I., Scherrer S.S. (2020). Could 3D printing be the future for oral soft tissue regeneration?. Bioprinting.

[86] Yang X.T., Ma Y., Wang X.T., Yuan S.M., Huo F.J., Yi G.Z., Zhang J.Y., Yang B., Tian W.D. (2023). A 3D-bioprinted functional module based on decellularized extracellular matrix bioink for periodontal regeneration. Adv. Sci..

[87] de Souza Araujo I.J., Perkins R.S., Ibrahim M.M., Huang G.T.-J., Zhang W. (2024). Bioprinting PDLSC-laden collagen scaffolds for periodontal ligament regeneration. ACS Appl. Mater. Interfaces.

[88] Liu J., Ruan J.P., Weir M.D., Ren K., Schneider A., Wang P., Oates T.W., Chang X.F., Xu H.H.K. (2019). Periodontal bone-liga-ment-cementum regeneration via scaffolds and stem cells. Cells.

[89] Hu Y., Zhu T., Cui H.T., Cui H.J. (2025). Integrating 3D bioprinting and organoids to better recapitulate the complexity of cellular microenvironments for tissue engineering. Adv. Healthc. Mater..

[90] Cadamuro F., Nicotra F., Russo L. (2023). 3D printed tissue models: From hydrogels to biomedical applications. J. Control. Release.

[91] Reyes M.G., Torras A.B., Carrillo J.A.C., García J.M.V., Aguilar J.J.C. (2022). A study of tensile and bending properties of 3D-printed biocompatible materials used in dental appliances. J. Mater. Sci..

[92] Jeong H.Y., Woo B.H., Kim N., Jun Y.C. (2020). Multicolor 4D printing of shapememory polymers for light-induced selective heating and remote actuation. Sci. Rep..

[93] Wan X., He Y., Liu Y.J., Leng J.S. (2022). 4D printing of multiple shape memory polymer and nanocomposites with biocompatible, programmable and selectively actuated properties. Addit. Manuf..

